# Assessing drivers of export orientation in the subsea oil and gas industry

**DOI:** 10.1186/s40064-015-1203-4

**Published:** 2015-08-08

**Authors:** Jarle Aarstad, Inger Beate Pettersen, Stig-Erik Jakobsen

**Affiliations:** Faculty of Engineering and Business Administration, Centre for Innovation, Bergen University College, PO Box 7030, 5020 Bergen, Norway

**Keywords:** Export orientation, Innovation, Internationalization, Value chain position, Piggybacking, Subsea oil and gas industry

## Abstract

The purpose of this short study was to identify the drivers of export orientation of firms in the subsea oil and gas industry in Western Norway. As the oil fields in the North Sea are approaching a stage of maturity, gaining knowledge of these drivers is crucial. An online survey was conducted of firms operating in the subsea oil and gas industry in the region. Consistent with previous research, the data reveal that product innovation and a majority share of international ownership increase firms’ export rates. The use of instrumental variables indicates that both product innovation and international ownership are *causes* of subsea petroleum exports. The study moreover finds that subcontractors have a lower rate of direct exports than system providers, but international ownership in particular boosts subcontractors’ export rates, probably by decreasing their market dependency on regional system providers. A clear recommendation for managers and stakeholders is that they should encourage foreign investments throughout the value chain. The results of such a strategy appear to be especially positive for subcontractors.

## Background

The focus of this short study is to identify the possible drivers of export orientation in the Norwegian maritime subsea oil and gas industry. The maritime subsea oil and gas industry can be broadly defined as the technological enterprises involved in developing, producing, implementing or operating devices and production systems for the extraction of oil and gas from the offshore seabed. In Norway, the subsea industry is located in several geographical regions; this paper focuses on the cluster of subsea firms located on the west coast of the region of Bergen, the country’s second largest city. Firms in the cluster perform maintenance, modifications and operations in subsea installations, as well as developing and producing innovative and cutting-edge technical products. In 2006, the subsea cluster gained the status of *Norwegian Centre of Expertise*, which is a national cluster programme intended to strengthen innovation, collaboration and internationalization in the most modern, sustainable and ambitious regional industrial clusters in Norway (http://www.nce.no/no/Om-NCE/About-NCE/). Locally and worldwide, and despite a recent decrease in the price of crude oil, the subsea industry has grown strongly. Yet as the North Sea oil fields approach a stage of maturity—indicating slower local growth—a further increase is expected to take place internationally. Accordingly, Norwegian subsea firms may have to adopt an export-orientated strategy to pursue long-term growth and continuous expansion.

Technologically, the Norwegian subsea industry is regarded as world-leading, and is a result of a long-term national strategy focusing on building a national supply industry, enhancing oil- and gas-related competence, R&D and technological innovation (Bjørnstad 2009). However, despite the regional industry’s technological leadership in its focal domains, there is strong variation in subsea firms’ export orientation. Some firms export almost all of their subsea-related production, whereas others export practically none.

Previous studies have demonstrated a strong link between firm innovation, international ownership and export orientation (e.g., Yi et al. 2013; Du and Girma 2009; Filipescu et al. 2013). The current study addresses these concepts, but it also applies instrumental variables in the estimates to assess whether product innovation and international ownership are genuine causes of export orientation. A novel contribution is the study’s focus on the position of subsea firms in the value chain (Porter 1985) and the possible implications for their export orientation. Therefore, it may be argued that the current contribution is relevant to strategic management in industries that share similarities with the local context studied here.

The outline of the paper is as follows. The next section discusses relevant theory and develops some testable hypotheses. The research context is then presented and the methodology for the statistical analyses is described. Next, hypotheses are tested and other empirical results are presented. The final section discusses the implications of the empirical findings for strategic management theory and practice, addresses the study’s limitations, and suggests avenues for future research.

## Theoretical positioning and hypotheses

Two studies conducted on the Aberdeen oil cluster, which is also approaching maturity, have addressed issues relevant to the subsea oil and gas cluster in the region of Bergen. Chapman et al. (2004) found that upstream specialist suppliers were more likely to favour a geographical diversification strategy, i.e., moving into overseas oil fields. In contrast, suppliers engaged in the more generic downstream activities of manufacturing, engineering or business services favoured sectorial diversification in the home country. Raines et al. (2001) found that key specialist technology suppliers to foreign investors in the oil sector acquired knowledge, networks and credibility through their investors that accelerated their internationalization process, both through piggybacking, i.e., indirect export through local system providers (cf. Terpstra and Chwo-Ming 1990), and eventually direct exports.

**Fig. 1:**
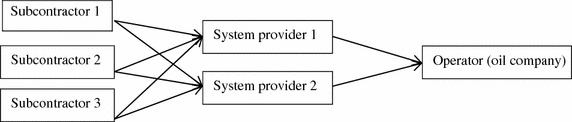
A simplified model of the value chain in the petroleum subsea industry.

The value chain (cf. Porter 1985) in the subsea industry is generally as follows (see Fig. 1): operating (oil) companies, system suppliers, and subcontractors (in addition to other enterprises, such as consultancy firms). The purpose of this study is to compare the export rate of subcontractors and system providers, and we have not found other studies that that explicitly examine this issue. System suppliers and subcontractors are particularly relevant to study since they develop, produce or implement devices and production systems that enable the extraction of oil and gas. In other words, the focus in this study is to examine system suppliers and subcontractors producing devices for the extraction of oil and gas. Such devices can be sold domestically or be exported to foreign markets.

It could be argued that subcontractors have constraints in terms of potential direct subsea exports, because their dependency on system providers prevents them from gaining an independent and genuine share of the growth of the worldwide subsea industry. Most commonly, subcontractors sell a large share of their production to a local system provider, which in turn is (partly) exported. This phenomenon of piggybacking in indirect exporting is a well-known practice in the oil and gas sector (Raines et al. 2001; Chapman et al. 2004). The piggybacking mode enables suppliers to enter overseas markets that they may not have managed to enter independently.

Nevertheless, it is important to emphasize that these relations are asymmetric power constellations between large and small firms (Echeverri-Carroll et al. 1998). For instance, Crabtree et al. (1997) found that supply chains in the Scottish oil and gas sector had been exacerbated by lock-in relations with large locally integrated contractors. In a similar vein, studying the Norwegian petroleum industry, Nilsson and Aarstad (2012, p. 314) state that small and medium-sized enterprises “lack capacity to collaborate in complex and large-scale projects with the supplying companies for the operators at the oil fields in the North Sea”. Consequently, it may be assumed that asymmetric and lock-in power relations, and dependency on system providers, limit subcontractors’ direct access to foreign markets. It is therefore likely that they have a lower rate of direct export than the system providers. In line with this reasoning, a study of the defence industry in Norway shows that system integrators that assemble a broad set of competences have higher export than specialists suppliers (Fevolden et al. 2014). We therefore propose the following hypothesis:

*Hypothesis 1* (*H1*) Subcontractors will have a lower rate of direct export than system providers.

The positive link between innovation and export rate has been demonstrated in a number of studies in different national contexts, such as China (Yi et al. 2013; Chen 2012), Spain (Filipescu et al. 2013; Cassiman and Golovko 2011; Monreal-Perez et al. 2012), Germany (Becker and Egger 2013) and the UK (Higon and Driffield 2011; Ganotakis and Lovey 2011). Innovative firms in particular seem to increase the export rate in competitive and dynamic markets (Boso et al. 2013). Despite the subcontractors’ liabilities in terms of direct exports, it may be argued that innovation strategies decrease dependency on system providers, because of the innovative firms’ attractiveness in international export markets.

Studies have further shown that local suppliers can benefit greatly from their foreign investors, which are typically multinational firms with an extensive pool of knowledge and skills, technologies, networks, market contacts and credibility effects (e.g. Young and Wilkinson 1989). Thus, it may be argued that an international distribution system and branding effects resulting from international ownership (Du and Girma 2009) will bypass subcontractors’ liabilities and boost their export rate. Ricci and Trionfetti (2012) have found that foreign ownership and foreign international linkages increased the export rate. Wang et al. (2013) have in a similar vein shown that acquiring technology from foreign markets boosted Chinese manufacturing firms’ export rate, and a French study found that offshore outsourcing increased the export (Bertrand 2011). These effects are probably a result of both increased international experience and a branding effect of being exposed to foreign markets. Another study from China has shown that the relationship between foreign domestic ownership and export orientation was particularly strong for innovating firms (Yi et al. 2013).

Taken together, it is surmised that an innovation strategy and international ownership will boost subcontractors’ export rates. The following hypotheses are proposed:

*Hypothesis 2a* (*H2a*) An innovation strategy will boost subcontractors’ export rate.

*Hypothesis 2b* (*H2b*) International ownership will boost subcontractors’ export rate.

## Research context and methodology

Candidate firms for this study were mainly recruited from the *Norwegian Centre of Expertise (NCE) Subsea Membership List*. As noted, the *NCE* is a national cluster programme intended to strengthen innovation, collaboration and internationalization in the most modern, sustainable and ambitious regional industrial clusters in Norway. In addition, other relevant firms in the region were identified by SIC codes obtained from Statistics Norway. Overall, 126 suitable candidate firms were identified. An electronic questionnaire was developed and submitted to the general managers of the candidate firms. Data were gathered during the final months of 2009 and the beginning of 2010.

The general managers were requested to respond to questions concerning the subsea petroleum activities of their firms. A total of 75 firms returned the questionnaire. We excluded one oil company that participated in the survey. Statistically, it can be problematic to compare one unit of observation with other observations in the data. And as we have noted, system suppliers and subcontractors are furthermore particularly relevant to study since they develop, produce or implement devices and production systems that enable the extraction of oil and gas. The majority of the statistical analyses had a sample size ranging from 65 to 70 firms.

The effect variable for this study, the firms’ export orientation or export rate, was measured by requesting the respondents to indicate the percentage of their turnover that was international (i.e., excluding domestic turnover) in the subsea segment, which could be: (1) none, (2) less than 10 %, (3) 10–50 %, or (4) more than 50 %. Product innovation was measured as a dummy variable by requesting the respondents to indicate whether the firm had developed new or substantially improved products or services during the previous 3 years (coded 1 if yes and 0 otherwise). The wording is similar to the methodology used by the European Innovation Scoreboard (2006). Other studies have applied a similar measure of firm innovation (e.g., Bertrand and Mol 2013).

International ownership was also measured as a dummy variable for which the respondents were requested to indicate whether the firm had a majority of international ownership or a majority of national ownership (coded 1 for international ownership and 0 for national ownership). Lately, foreign technology and oil service companies have acquired a number of companies in the subsea industry cluster in the Bergen region. Motives for these investments have been to strengthen their market position in the North Sea and to acquire knowledge about subsea technologies that have been developed in the region. To cite a few examples; the globally leading technology and process management company, Emerson, acquired the local company Roxar in 2009, the large multinational oil service company, Schlumberger, acquired the local company Framo Engineering in 2011, and the world leading energy supplier, Siemens, acquired the local company Bennex Group in 2011.

Position in the value chain was also measured as a dummy variable; subcontractors and “other firms” were coded 1, and system providers were coded 0 (default value). The study controlled for firm size in terms of number of employees. In unreported models, the study also controlled for collaboration with customers, suppliers and R&D institutions, whether firms were part of a corporation, and recruited internationally or locally. However, these concepts had no genuine effect on export orientation and did not alter the overall conclusions of this study. Accordingly, estimates with these variables were not included in this paper. (The data are available from the first author upon request).

## Results

### Correlations and ordinary least squares regressions

The correlation matrix shown in Table 1 shows that the subsea export rate correlates with innovation and majority international ownership (which is consistent with the findings reported below). Table 2 reports ordinary least squares (OLS) multiple regressions where the subsea firms’ export rate is modelled as a dependent variable. An advantage of OLS multiple regression is that it allows us to explicitly “control for many other factors (beyond the factors that we are genuinely interested in) that simultaneously (may) affect the dependent variable” (Wooldridge 2006, p. 73). Another advantage is that it calculates the explained variance of the regression model (R-square), in addition to calculating adjusted explained variance (Adj. R-square) that also takes account of the number of independent variables in the model (for further explanations, see for instance Wooldridge 2006, pp. 85–87, 208–209).

**Table 1:** Correlation matrix

Mean	SD		1	2	3
2.04	1.06	Subsea export (1)			
3.08	1.44	Firm size (2)	0.188		
0.686	0.468	Innovation (3)	0.385**	0.090	
0.141	0.350	Majority international ownership	0.252*	−0.170	−0.005

N = 68–71.

* p < 0.05, ** p < 0.01, two-tailed tests.

**Table 2:** Ordinary least squares regressions

	Model 1	Model 2	Model 3	Model 4	Model 5	Model 6	β coef.	Model 7	β coef.
Intercept	1.618*** (0.297)	2.715*** (0.462)	1.419*** (0.312)	2.444*** (0.462)	2.384*** (0.480)	3.238*** (0.578)		2.148*** (0.420)	
Firm size	0.138 (0.087)	0.117 (0.083)	0.165^†^ (0.088)	0.158^†^ (0.083)	0.084 (0.090)	0.109 (0.083)	0.148	0.166* (0.081)	0.228
Innovation		0.813** (0.254)		0.817** (0.252)		0.886*** (0.251)	0.389	0.829** (0.240)	0.395
Majority international ownership			0.868* (0.354)	0.923** (0.323)		0.983** (0.309)	0.342	1.397*** (0.294)	0.534
Subcontractors					−0.756* (0.359)	−0.773* (309)	−0.349		
Other firms					−0.538 (0.443)	−0.236 (0.408)	−0.082	0.557^†^ (0.288)	0.222
R-square	0.035	0.174	0.112	0.264	0.097	0.351		0.417	
Adj. R-square	0.021	0.148	0.085	0.227	0.056	0.296		0.369	
F value	2.49	6.72**	4.09*	7.28***	2.36^†^	6.38***		8.75***	
N	70	67	68	65	70	65		54	

Regression estimates with standard error in parentheses. Dependent variable: subsea export.

^†^p < 0.10, * p < 0.05, ** p < 0.01, *** p < 0.001, two-tailed tests.

It can be seen that firm size, as a control variable, is positively associated with subsea export rate in all models reported, but the effects are non-significant or borderline significant in most models. More importantly, innovation and a majority of international ownership are significantly associated with the subsea export rate in all models that include the parameters (of innovation and a majority of international ownership). These findings are consistent with previous research (e.g., Yi et al. 2013; Du and Girma 2009; Filipescu et al. 2013), and thus increase the criterion validity of the current study (cf. Cronbach and Meehl 1955).

All regression estimates with a significance level (p value) of less than 0.05 implies a confidential level that is higher than 95 %. In other words, if a regression estimate has a significance level of 0.05 or lower we can be at least 95 % sure that the independent variable is either positively or negatively associated with the dependent variable. If the significance level is lower than 0.01 we can be at least 99 % sure. Taken together, the lower the significance level (p value) the higher the confidence interval. A low significance level is accordingly indicative of a robust regression estimate (for further readings and assumptions about confidential intervals in statistics and econometrics, see for instance Wooldridge 2006).

Models 5 and 6 in Table 2 show that subcontractors have a significantly lower rate of export than system providers (p = 0.015 in Model 6), which supports H1 (system providers are coded with a default value of 0, so they do not appear in the table). In Model 7, system providers are omitted from the data, and it can be seen that the effect size of international ownership increases substantially (compared with Model 6), whereas the effect of innovation is mostly the same. This indicates that international ownership in particular boosts subcontractors’ export rates (but innovation does not). It can therefore be concluded that H2b has empirical support, whereas H2a is rejected.

Ideally, the model should include an interaction term between the concept of subcontractor (vs. system provider) and innovation to test H2a. Likewise, there should be an interaction term between the concept of subcontractor (vs. system provider) and majority international ownership to test H2b. This has been done in unreported models, and the results are in line with those reported so far: H2b gains strong and significant empirical support whereas H2a is rejected. However, we have only ten observations for system providers in our data, which may hamper the statistical validity of modelling interaction terms with so few observations. Nevertheless, consistency in the empirical results indicates overall support for H2b and a rejection of H2a. (Statistical details of the regressions with these interaction terms are available from the first author upon request).

### Ordinal logistic regressions and estimations with instrumental variables

We have argued that ordinary least squares (OLS) multiple regression has several advantages, but a limitation is a requirement of a normally distributed dependent variable (see Wooldridge 2006, Ch. 4). Since the dependent variable of export rate was measured crudely according to four classes (none export, less than 10 % export, 10–50 % export, and more than 50 % export), this may violate the requirement of a normally distributed dependent variable. The dependent variable may instead take a property of an ordinal (or ordered categorical) distribution.

To take account of this, Models 6 and 7 in Table 2 were replicated with estimations of ordinal logistic regressions instead of OLS regressions. An advantage of using ordinal logistic regression is that it efficiently and correctly calculates independent variables when the dependent variable takes an ordinal (or ordered categorical) distribution (for further readings about ordinal logistic regression, see for instance Kleinbaum and Klein 2010). Data analyses with ordinal logistic regressions are reported in Table 3, and the results are in line with those reported for Models 6 and 7 in Table 2. Comparing regression estimates between OLS regression and ordinal logistics regression is not straightforward, but significance levels and also the signs (positive or negative) between different estimates in Tables 2 and 3 are very consistent.

**Table 3:** Ordinal logistic regressions

	Model 6	Model 7
Intercept 1	−3.603 (1.372)	−1.276 (1.130)
Intercept 2	−2.096 (1.331)	0.405 (1.137)
Intercept 3	−0.185 (1.296)	2.516 (1.212)
Firm size	0.276 (0.192)	0.446^†^ (0.224)
Innovation	2.269*** (0.655)	2.419** (0.753)
Majority international ownership	2.524** (0.773)	3.337*** (0.863)
Subcontractors	−1.896* (0.740)	
Other firms	−0.543 (0.890)	1.548^†^ (0.804)
Chi square	29.73***	27.71***
N	65	54

Regression estimates with standard error in parentheses. Dependent variable: subsea export.

^†^p < 0.10, * p < 0.05, ** p < 0.01, *** p < 0.001, two-tailed tests.

We have referred to advantages of using multiple ordinary least square (OLS) and ordinal logistic regression. However, a liability of these two techniques is that they cannot rule out the possibility of potential reverse orders of causality. For instance, firms with a high export rate may have an increased possibility of product innovation, because of factors such as access to novel and non-redundant information in foreign markets, or a push to become innovative in order to face the challenges of strong international competition in foreign markets. Likewise, firms with an initially high export rate may be considered favourable candidates for foreign investors. To address these issues of possible reverse orders of causality, we conducted estimations with instrumental variables using Stata 13 (StataCorp 2013). An advantage of using estimations with instrumental variables is that it can enable us to take account of potential reverse orders of causality (see for instance Wooldridge 2006, Ch. 15). Valid instrumental variables are correlated with the independent variable, but uncorrelated with the error term of the estimates (ibid.).

Different techniques can be used carry out estimations with instrumental variables, but in this study, we use generalized method of moments (GMM) estimations, developed by Hansen (1982). A particular advantage of this technique is that is robust to potential heteroskedasticity (ibid.). Table 4 reports GMM estimations with instrumental variables. In Model 1, dummies for collaboration in terms of product development with customers and suppliers are the instruments and innovation is instrumented. In Model 2, the dummy for being part of a corporation and tendency towards local versus international recruitment are instruments, and majority international ownership is instrumented. Estimation with the instrumental variables in Model 1 shows that innovation is a significant predictor of export orientation. Likewise, estimation with instrumental variables in Model 2 shows that a majority of international ownership is a significant predictor of export orientation.

**Table 4:** Generalized method of moments estimates with instrumental variables

	Model 1	Model 2
Intercept	3.671*** (0.561)	1.640*** (0.145)
Innovation	1.269** (0.423)	
Majority international ownership		1.846** (0.583)
Wald Chi square	8.99**	10.03**
First-stage regression partial R-square	0.336	0.376
F value	15.24***	11.84***
Hansen’s *J* Chi square (p value in parentheses)	0.603 (0.438)	0.045 (0.831)
N	67	57

Regression estimates with robust standard errors in parentheses. Dependent variable: subsea export.

* p < 0.05, ** p < 0.01, *** p < 0.001, two-tailed tests.

Hansen’s (1982) *J* Chi square is insignificant in both models, which indicates that the instruments are uncorrelated with the error term. A significant first-stage regression partial R-square shows that the instruments are correlated with innovation and majority international ownership. Both these tests, showing that the (1) instruments are correlated with the independent and instrumented variables and (2) uncorrelated with the error term, indicate that we have used valid instruments in our estimations (for a further discussion of the validation of instruments, see, e.g., Stock et al. 2002). We therefore concluded that product innovation and a majority of international ownership genuinely cause an increase in subsea firms’ export rate.

A potential limitation with the use of instrumental variables is that “poor” instruments, that are either weakly correlated with the independent variable and/or correlated with the error term, will result in incorrect or inefficient regression estimates (Wooldridge 2006, Ch. 15). In the current study, we have tested the validity of the instruments and have found them to be appropriate in our estimates. We also observe that the statistical conclusions of our estimations with instrumental variables are in line with the OLS regressions and the ordinal logistic regressions. It is finally worth noting that the regression estimates reported in Table 4 with the use of appropriate instrumental variables are higher than those reported by the use of OLS regressions in Table 2. This indicates that OLS regressions in fact tend to underreport the de facto effects of innovation and a majority of international ownership as drivers of export orientation.

A particular limitation with the use of GMM estimations with instrumental variables is that a relatively low sample size can underestimate regressors that would have been significant with the use of other techniques (Hayashi 2000). GMM is thus a conservative technique in current study (due to its relatively low sample sizes). Observing significant effects in Table 4 is accordingly indicative of robust statistical results.

## Discussion and conclusion

The purpose of this short study was to identify the drivers of export orientation of firms in the subsea petroleum industry in Western Norway. As the oil fields in the North Sea are approaching a stage of maturity, gaining knowledge of these drivers is crucial.

### Theoretical implications

Consistent with previous research, the data reveal that product innovation and a majority share of international ownership increase firms’ export rates. The use of instrumental variables indicates that both product innovation and international ownership are causes of export orientation. The data furthermore show that subcontractors have a lower rate of direct export than system providers, which is a novel contribution. It thus appears that subcontractors have constraints in terms of potential subsea export. Subcontractors most likely sell a large share of their production to local system providers, and this in turn is (partly) exported, a practice known as piggybacking (Raines et al. 2001; Chapman et al. 2004). However, subcontractors’ apparent dependence on system contractors may also constrain them from gaining an independent and genuine share of the growth of the worldwide subsea industry.

Some regression models omitted the system providers from the data, and we find that international ownership in particular boosted subcontractors’ export rate. This is also a novel contribution, and the observed effect is probably a result of a decrease in subcontractors’ market dependency on regional system providers when they have international ownership. International ownership thus appears to boost the export rate for subcontractors in particular, most likely as a function of access to international distribution channels and branding effects through international ownership. Innovative subcontractors also tended to have an export orientation, but this effect was not found to be particularly strong for this group of firms (compared with system providers).

### Practical implications, limitations and avenues for future research

To achieve a larger market share for the expanding international subsea industry, local firms should pursue innovative strategies, but subcontractors in particular should aim to increase their attractiveness to international investors to increase their lagging direct subsea exports. Therefore, a clear recommendation for managers and stakeholders in this industry is to encourage foreign investments throughout the value chain, because this appears to have an especially positive effect for subcontractors. That said, innovation strategies are not cost-free and also entail uncertain outcomes; thus, potential costs and risks should be taken into account. It should also be considered whether a local firm has any innovative potential, and it is probable that marginal costs and risks of introducing innovative products and services may differ between firms that operate in the industry. Although majority international ownership appears to be advantageous with reference to an export orientation, we briefly address later the potential downsides of pursuing such a strategy.

A limitation of this study is its relatively small sample size. Therefore, future contributions should aim to replicate the study using data from a larger number of firms, most preferably in longitudinal research over a time span. In particular, the proportion of system providers is low in our sample. Nevertheless, the statistical effects are strong and consistent across different regression estimates. The use of instrumental variables furthermore indicates that innovation strategies and international ownership are genuine causes of subsea firms’ export orientation.

To increase external validity, researchers should replicate the methodology in industries that share similarities with the petroleum sector. To scrutinize subcontractors’ liabilities further in terms of direct exports, triangulation of methodologies should be encouraged, for instance, by combining quantitative with qualitative data.

Recently, the petroleum industry and society at large have observed a decrease in the price of oil. The long-term effects are still unclear, but future research should aim to investigate this issue with reference to subsea firms’ innovative strategies, international versus domestic ownership structure and export orientation. For instance, will firms focus on cost control at the expense of innovation strategies, and is this likely to influence the relationship between innovation and export orientation? Future research should also study whether there are any long-term effects of international ownership. It is probable that the short-term effects are positive if there is a boost in export rate as a function of a branding effect and access to overseas markets through international distribution channels. However, the long-term effects may be less positive if the mother company starts to downsize or outsource local activities. This may be especially relevant when the oil price is low and the focus on reducing costs is overt and prevalent.
